# Depression and perceived stress among perinatal women living with HIV in Nigeria

**DOI:** 10.3389/fpubh.2023.1259830

**Published:** 2023-11-20

**Authors:** Folahanmi T. Akinsolu, Olunike R. Abodunrin, Abisola A. Lawale, Samuel A. Bankole, Zaniab O. Adegbite, Ifeoluwa E. Adewole, Mobolaji T. Olagunju, Oluwabukola M. Ola, Anita M. Dabar, Rukayat A. Sanni-Adeniyi, Aisha O. Gambari, Diana Wangeshi Njuguna, Abideen O. Salako, Oliver C. Ezechi

**Affiliations:** ^1^Department of Public Health, Lead City University, Ibadan, Nigeria; ^2^Nigerian Institute of Medical Research, Lagos, Nigeria; ^3^Lagos State Health Management Agency, Ministry of Health, Lagos, Nigeria; ^4^Department of Epidemiology and Health Statistics, School of Public Health, Nanjing Medical University, Jiangning, Jiangsu, China; ^5^School of Nursing, Department of Medical-Surgical Nursing, Dedan Kimathi University of Technology, Nairobi, Kenya

**Keywords:** depression, stress, HIV, pregnancy, mental health

## Abstract

**Background:**

Pregnancy and postpartum periods are crucial stages for women’s mental health, and women living with HIV are particularly susceptible to depression and psychological stress due to various factors. This study investigated the prevalence and associated factors of depression and perceived stress among women living with HIV during their perinatal period in Ibadan, Nigeria.

**Methods:**

A cross-sectional survey was conducted in three HIV treatment centers in Ibadan, Nigeria, among women living with HIV between the ages of 19 and 49 who were either pregnant or had given birth within the last 2 years. The study was conducted from September 2022 to December 2022. An interviewer-administered questionnaire was used to collect the data from the participants. Ethical approval and informed consent were obtained, and data were analyzed using the Statistical Package for Social Science version 26.

**Results:**

The study included 402 participants, of whom 69.0 and 78.0% reported symptoms of depression and perceived stress, respectively. However, 15.2% of the participants have comorbid depression and stress. Positive partner status was significantly associated with lower perceived depression, while gestational age between 29 and 40 weeks was significantly associated with lower perceived stress. The co-occurrence of depression and perceived stress was associated with partner status, income level, family support, gestational age, and years on antiretroviral therapy.

**Conclusion:**

The high prevalence of depression, perceived stress, and their co-occurrence among women living with HIV during the perinatal period call for incorporating mental health care into routine maternal healthcare for all women, particularly those living with HIV. This finding emphasizes the need for public health efforts to prioritize perinatal mental health and improve access to care and support for women and their partners.

## Introduction

For decades, the HIV epidemic has remained a significant global health challenge, with an estimated 38 million individuals infected globally. Sub-Saharan Africa accounts for 71% of the worldwide population of people living with HIV (1), with a prevalence rate of 1.4% in Nigeria, making it the third most HIV-burdened country (1). Moreover, the burden of HIV in Nigeria is the highest among the female adult population and a known predisposition of maternal mortality with an estimated prevalence of 26.4% among pregnant cohorts (2, 3).

More women have symptoms of depression in comparison to men, and this gendered pattern exists among people living with HIV (4–6). Women living with HIV (WLHIV), especially in low and middle-income countries (LMICs), experience significant psychological challenges, such as depression, stress, and anxiety, due to their HIV diagnosis (7, 8). Studies have also shown that WLHIV is susceptible to more severe mental illness symptoms such as depression, anxiety, and posttraumatic stress disorder (6, 9).

Pregnancy and postpartum periods are some of the most vulnerable periods that may contribute to symptoms of depression in women (10). The prevalence of depressive symptoms among pregnant women ranges from 11.4 to 40.0%, which is higher than that of women generally (11, 12). In a study conducted in Nigeria, the prevalence of postpartum depression was 35.6% (13), while in Kenya, 44.2% (14) was reported among other locations where similar studies have been conducted. Furthermore, as previously reported in a study, mothers who experienced preterm deliveries had 5.75 times higher odds of developing symptoms associated with depression and anxiety compared to mothers who carried their pregnancies to full term. This elevated risk can be attributed to the unexpected nature of premature childbirth, catching the mothers off guard and leaving them unprepared for the delivery at that specific moment (14). Women’s brains change structurally, psychologically, and behaviorally throughout pregnancy as they prepare for their new role as mothers (15). These changes, however, make pregnant women more prone to stress (16), which increases the likelihood of developing prenatal depression and stress symptoms (17).

Pregnancy can increase psychological susceptibility to WLHIV due to environmental factors, disclosure concerns, and HIV-related stigma (18). Studies conducted in LMICs have found that pregnant and postpartum WLHIV suffer from a high prevalence of depression and psychological stress (19–21). Similarly, a systematic review in Africa examined the prevalence of perinatal depression in HIV-infected women. The weighted mean prevalence of antenatal and postnatal depression was 23.4 and 22.5%, respectively (22). Depression is also associated with adherence to care and therapy among pregnant WLHIV (21), which may result in treatment failure and increased vertical HIV transmission (7). Psychological issues such as depression and stress may also adversely affect obstetric and neonatal outcomes and increase the risk of mother-to-child transmission (23).

Women, particularly in developing countries, are more likely to be exposed to risk factors such as poor socioeconomic status, making them more susceptible to perinatal depression (24). Depression and psychological stress may be critical barriers to HIV treatment and prevention as the conditions may be linked. In addition, women newly diagnosed with HIV during pregnancy and receiving a positive HIV diagnosis can generate worry and fear of transmitting the virus to an unborn child (25).

**Figure 1:**
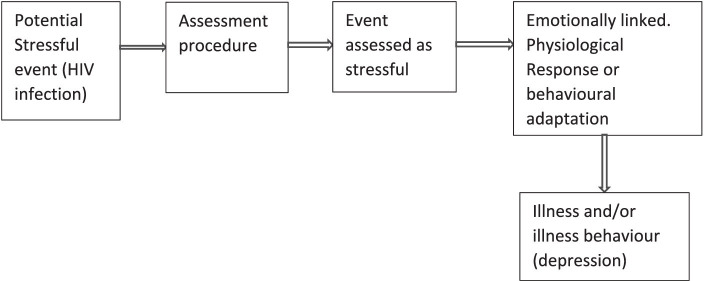
Conceptual framework on the relationship among HIV-related stress and depression among WLHIV (26).

According to the Cohen and Wills Framework, the relationship between HIV-related stress and depression was assessed (26), focusing on the interplay between stress, social support, and their impact on an individual’s well-being. The authors introduced the buffering hypothesis, which suggests that social support can act as a protective factor against the adverse effects of stress on an individual’s mental and physical health (26). Hence, the framework explores how a strong social support system can mitigate the adverse consequences of stress, ultimately contributing to better overall psychological and physiological outcomes. This conceptual framework has been influential in understanding the role of social support in coping with various stressors and its implications for health and well-being (Figure 1) (26).

Pregnancy is a significant life change and can affect mental health, and the specific challenges WLHIV faces may exacerbate these effects. Therefore, it is essential to consider their holistic situation when providing care and support (20). Therefore, understanding the magnitude of depression and stress, as well as their associated factors, among perinatal WLHIV could provide important information that could aid in mitigating the poor mental health experienced by this group of women. This study is among the few studies investigating the prevalence of depression and psychological stress among WLHIV during pregnancy and the postpartum period in Nigeria. Thus, the study aims to determine the prevalence and factors associated with depression and psychological stress among WLHIV during their perinatal period in Ibadan, Nigeria, and to assess the relationship between HIV-related stress and depression among WLHIV.

## Materials and methods

### Study design and setting

This study was a facility-based cross-sectional survey conducted in three HIV treatment centers in Ibadan. The three centers providing HIV treatment were randomly selected from the twelve accredited ART facilities in Ibadan. The centers were State Hospital, Adeoyo, Ringroad; Adeoyo Maternity Health Centre; and St Annes Anglican Hospital, Molete. These health centers provide comprehensive HIV services and antenatal, delivery, and postnatal care for WLHIV in Ibadan, Oyo State.

### Study population

The study population included women of known HIV status aged 18 years and above who were pregnant or had given birth within the last 2 years and receiving treatment at any of the three selected antiretroviral treatment centers in Ibadan, Oyo State. The women were consecutively selected over 5 weeks at the selected HIV treatment facilities. Women who were unable to provide informed consent were excluded from the study.

### Sampling method and sample size determination

The sampling method for this study was convenience sampling, a type of non-probability sampling technique. The study sample size was calculated according to the formula *N* = *Z*α^2^*P* (1 − *P*)/*d*^2^, where *Z*α is the *Z* statistic for a 95% confidence level, *N* is the sample size, *P* is the prevalence of WLHIV, and d is the precision. Using a confidence level of 95%, a precision level of 5%, and a design effect of 1.5, we calculated a minimum sample size of 384. We increased the sample size to 402 for potential missing data and non-response.

### Data collection tool

The study used an interviewer-administered questionnaire to gather data on various demographic variables of the participants, including their age, level of education, primary occupation, and average monthly *per capita* income. In addition, the study questionnaire collected information on depression using the Edinburgh Postnatal Depression Scale (EPDS) (27, 28) and perceived stress using the Perceived Stress Scale-10 (PSS-10) (29, 30).

The EPDS was used to assess symptoms of perinatal depression. The scale has ten items with responses on a 4-point Likert scale ranging from 0 (absence of depressive moods) to 3 (worst mood). A score ranging from 0 to 30 is calculated, and a cut-off point of ≥12 indicates an increased likelihood of clinical depression (31). The scale does not mention pregnancy, child, birth, or infant and has also been validated in a non-pregnant population (31).

Perceived stress was measured using the Perceived Stress Scale. The Perceived Stress Scale (PSS) is a 10-item (32) self-report assessment of the stress domains of unpredictability, lack of control, burden overload, and stressful life circumstances. Responses are on a 5-point Likert scale ranging from 0 (never) to 4 (very often) (32). The PSS score is the sum of all responses, with higher scores indicating more perceived stress and can range from 0 to 40.

Before the main study, the EPDS and PSS-10 were pretested on a representative sample. Feedback on clarity and relevance was collected, and statistical analyses, including Cronbach’s Alpha, affirmed their strong internal consistency (EPDS: α = 0.85; PSS-10: α = 0.83) and validity, ensuring their suitability for the research objectives.

### Data collection procedures

The data collection was conducted between September 2022 and December 2022. All research assistants were trained before the commencement of the study on the research tools, interviewing skills, data management, and clarifications of ethical issues in research. The research assistants administered the questionnaires in English or the local language to participants who could neither read nor write.

Years on ART and gestational age were orally taken from the participants and confirmed from their case files with the healthcare providers at the clinic. The participants filled in partner status and Status disclosure to the partner, and it was confirmed from their case files if they had reported the partner status to the clinic.

The questionnaires were administered privately, and clarification and assistance were provided where necessary. The interviews took approximately 20 min to complete.

### Statistical analysis

The data collected from the questionnaires were entered into Statistical Package for Social Science version 25 (SPSS 25) software for analysis. The demographic information of the study participants was summarized using descriptive statistics. Means and standard deviations were used to summarize continuous variables, while categorical variables were summarized using frequencies and percentages. The prevalence of depression and perceived stress were calculated as the proportion of participants who scored above the cut-off points on the EPDS and PSS-10 scales, respectively. Regression analyses were performed based on the categories of exposure and outcome variables. A logistic regression model was used when the outcome variable was dichotomized to determine the association between demographic, clinical, and pregnancy-related factors and depression and perceived stress. Crude models were made without any adjustments for covariates, and adjusted models were made similarly; all adjusted models were adjusted for age, sex, level of education, marital status, drinking, and smoking status, among other covariates. Independent variables were selected based on relevance to research questions, theoretical justification, and multicollinearity check. The level of significance was set at *p* < 0.05. Odds ratios (OR) and 95% confidence intervals (CI) were used to report the strength of the association between predictor variables and the outcomes of interest.

### Ethical considerations

This study was conducted following the ethical principles of the Declaration of Helsinki. Ethical approval was obtained from the Lead City University Health Research and Ethics Committee (LCU-REC/22/125) and the Oyo State Ministry of Health Research Ethics Committee (AD 13/479/44539). Before administering the questionnaire, the participants were given information sheets outlining the study’s objective and scope, which were duly explained to the participants in English or the local dialect (Yoruba/Pidgin). The participants were informed that participation in the study was voluntary and that they were free to withdraw from the study at any point without any consequences. The confidentiality and anonymity of the participants were ensured, and all data were kept confidential and were used only for research purposes. The participants were assured that participation or non-participation would not affect their access to healthcare services. In addition, participants who required psychological support after the study were referred to the appropriate healthcare professionals. The participants provided their written informed consent to participate in this study.

## Results

### Sociodemographic characteristics of participants

A total of 402 participants were eligible for this study, and the mean age of the study participants was 35.8 ± 6.60 years. 225 (56.0%) participants identified as Christians, 92.3% were married, and 352 (87.6%) were from the Yoruba tribe. 173 (43.0%) participants had attained a secondary school education, 334 (83.1%) were employed, and 234 (58.2%) earned an income below 20,000.00 NGN (<26.51 USD) (see Table 1).

**Table 1:** Sociodemographic characteristics of study participants (*n* = 402).

Characteristics	Total *n* = 402	Antenatal (*n* = 263) *N* (%)	Postpartum (*n* = 139) *N* (%)
**Age (*n* = 402)**			
Mean (S.D)	35.8 ± 6.60		
**Religion**			
Christianity	225	150 (57%)	75 (54%)
Islam	176	113 (43%)	64 (46%)
**Tribe**			
Yoruba	352	225 (85.6)	127 (91.4)
Igbo	29	21 (8)	8 (5.8)
Hausa	20	16 (6)	4 (2.9)
Others	1	1 (0.4)	0
**Level of education**			
Primary level	94	53 (20.2)	41 (29.5)
Secondary level	173	107 (40.7)	66 (47.5)
Tertiary level	90	68 (25.9)	22 (15.8)
None	45	35 (13.3)	10 (7.2)
**Marital status**			
Married	371	246 (93.5)	125 (89.9)
Divorced	9	6 (2.3)	3 (2.2)
Widowed	8	7 (2.7)	1 (0.7)
Separated	8	4 (1.5)	4 (2.9)
Single	6	0	6 (4.3)
**Type of partner**			
Spouse	373	247 (93.9)	126 (90.6)
Steady	11	7 (2.7)	4 (2.9)
Casual	10	7 (2.7)	3 (2.2)
None	8	2 (0.8)	6 (4.3)
**Employment status**			
Employed	334	225 (85.6)	109 (78.4)
Unemployed	68	38 (14.4)	30 (21.6)
**Income level**			
<20,000	234	157 (59.7)	77 (55.4)
20,000–30,000	48	19 (7.2)	29 (20.9)
31,000–40,000	77	60 (22.8)	17 (12.2)
41,000–50,000	27	13 (4.9)	14 (10.1)
>51,000	16	14 (5.3)	2 (1.4)

### Relationship and support-related, clinical, and pregnancy-related characteristics of participants

Of the 402 participants, 335 were aware of their partner’s status, with 152 having positive partners. 246 (61.2%) had disclosed their status to their partners, 318 (79.1%) reported receiving adequate support from their partners, and 287 (71.4%) reported receiving support from family and friends. 263 (65.4%) were pregnant with a gestational age range of 5–40 weeks. Only 73 (18.2%) women reported that the pregnancy was planned (see Table 2).

**Table 2:** Support and pregnancy-related characteristics.

Characteristics	Total	Antenatal (*n* = 263) *N* (%)	Postpartum (*n* = 139) *N* (%)
**Partners status(*n* = 402)**			
Positive	157	118 (44.9)	39 (28.1)
Negative	245	145 (55.1)	100 (72.0)
**Status disclosure to partner (*n* = 402)**			
Yes	246	153 (58.2)	93 (66.9)
No	156	110 (41.8)	46 (33.1)
**Support from partner**			
Yes	318	218 (82.8)	100 (72)
No	76	43 (20.9)	33 (23.8)
Not applicable	8	2 (0.8)	6 (4.3)
**Support from other family and friends**			
Yes	287	204 (77.6)	83 (59.7)
No	115	59 (22.4)	56 (40.3)
**History of conflict with partner**			
Yes	128	80 (30.4)	48 (34.5)
No	274	183 (68.8)	91 (65.5)
**Years on ART(*n* = 402)**			
≤ 1 Year	85	50 (19)	35 (25.2)
≤ 5 Years	194	136 (51.7)	58 (41.7)
>5 Years	77	46 (17.5)	15 (10.8)
> 10 Years	46	31 (11.8)	31 (22.3)
**Problems in previous pregnancy**			
Yes	175	136 (51.7)	39 (28.1)
No	227	127 (48.3)	100 (71.9)
**Planned pregnancy (*n* = 263)**			
Yes	73	73 (27.8)	N/A
No	190	190 (72.2)	N/A
**Gestational age (*n* = 263)**			
5–13 weeks	109	109 (41.4)	N/A
14–28 weeks	85	85 (32.3)	N/A
29–40 weeks	69	69 (26.3)	N/A

### Prevalence of depression and perceived stress among WLHIV

Of the antenatal respondents, 154 (58.6%) reported low stress, while moderate stress was reported among 55.0 (20.9%) antenatal respondents, while high stress was reported among 54.0 (20.5%). Of most postpartum respondents, 80.0 (57.6%) reported moderate stress, and perceived stress was significantly associated with pregnancy status (see Tables 3, 4). The prevalence mean score of postpartum respondents with stress is 14.41 (±7.163), while the prevalence mean score of antenatal respondents with stress is 12.27 (±9.757; *p* < 0.05) (see Table 3).

**Table 3:** Prevalence of respondents with stress and depression among WLHIV.

Variable		Total *N* (%)	Antenatal (*n* = 263) *N* (%)	Postpartum (*n* = 139) *N* (%)	*p* value
Stress	Mean (SD)		12.27 (±9.757)	14.41 (±7.163)	
	Low stress	206 (51.2)	154 (58.6)	52 (37.4)	0.000*
	Moderate stress	135 (33.6)	55 (20.9)	80 (57.6)	
	High perceived stress	61 (15.2)	54 (20.5)	7 (5.0)	
Depression	Mean (SD)		9.26 (±7.978)	13.32 (±6.479)	
	Without depressive symptoms	145 (36.1)	117 (44.5)	28 (20.1)	0.000*
	With depressive symptoms	257 (63.9)	146 (55.5)	111 (79.9)	

*Values significant at a *p* < 0.05.

**Table 4:** Association between depression and perceived stress.

Variable	Overall depression	Depression		*p*-value
		Antenatal depression *N* (%)	Postpartum depression *N* (%)	
Low stress	65	39 (60)	26 (40)	0.000*
Moderate stress	131	53 (40.5)	78 (59.5)	
High perceived stress	61	54 (88.5)	7 (11.5)	

*Values significant at a *p* < 0.05.

Among the antenatal respondents, 117 (44.5%) were without depressive symptoms, while a majority of them, 146 (55.5%) were found to be with depressive symptoms. Of the postpartum respondents, 28 (20.1%) respondents were without depressive symptoms, while a more significant proportion of 111 (79.9%) respondents exhibited depressive symptoms (see Table 3). Hence, it was found that the mean scores of the postpartum group is 13.32 (±6.479) and the antenatal group is 9.26 (±7.978; see Table 3). These results indicate a high prevalence of depressive symptoms among the respondents, with a higher proportion of respondents experiencing depressive symptoms in the postpartum period compared to the antenatal period.

A statistical association was observed between the level of stress and depression, and it was observed to be statistically significant at a *p*-value of 0.000. A high stress level was reported among the pregnant respondents who were reported to have depression symptoms (see Table 4). Furthermore, 61 (15.2%) respondents have a comorbidity of depression and stress.

### Prevalence of respondents with suicidal thought

The result shows that among respondents without depressive symptoms, a small proportion of 3.00 (2.10%) individuals reported never having suicidal thoughts, while the majority of 133 (91.7%) respondents reported experiencing suicidal thoughts quite often. Only 1.00 (0.7%) respondents reported hardly ever having such thoughts, and 8.00 (5.5%) individuals reported experiencing suicidal thoughts sometimes.

In contrast, among respondents with depressive symptoms, 138 (53.7%) respondents reported never having suicidal thoughts. A smaller percentage of 52.0 (20.2%) individuals reported hardly ever experiencing such thoughts, and 57.0 (22.2%) respondents sometimes reported suicidal thoughts. Interestingly, only 10 (3.9%) individuals with depressive symptoms reported experiencing suicidal thoughts quite often.

### Factors associated with depression among WLHIV

Assessing the factors associated with depression among WLHIV, the crude OR of some of the factors were reported to be significant factors that affect the depression level among WLHIV (see Table 5). However, after adjusting for other factors, age ranges between 25 and 30 years, income level below 40,000 NGN (51.58 USD) monthly, history of conflict with a partner, early and late gestational age problems with previous pregnancy, smoking, and alcohol usage and status disclosure were reported to remain significant factors that affect the depression level among WLHIV. Age (25–30 years), problems with a previous pregnancy, and smoking were the risk factors for the development of depression among WLHIV in Nigeria with OR = 3.324 (CI = 1.185–9.320), OR = 2.851 (CI = 1.546–4.260), and OR = 2.650 (CI = 1.038–6.771) respectively while other significant factors were observed to be protective (see Table 5).

**Table 5:** Factors associated with depression among women living with HIV.

Variables	Category	Crude OR 95%CL (lower-upper)	*p*-value	Adjusted OR 95% CI (lower-upper)	*p*-value
Age	19–24	9.706 (1.182–79.670)	0.034**	7.849 (0.745–82.640)	0.086
	25–30	1.594 (0.825–3.079)	0.165	3.324 (1.185–9.320)	0.022**
	31–36	1.908 (1.01–3.571)	0.043**	2.366 (0.895–6.259)	0.083
	37–42	1.458 (0.790–2.690)	0.228	1.659 (0.661–4.163)	0.281
	43–49	Ref		Ref	
Income level	<20,000	0.335 (0.074–1.512)	0.155	0.119 (0.016–0.882)	0.037**
	20,000–30,000	0.169 (0.035–0.825)	0.028**	0.108 (0.013–0.904)	0.040**
	31,000–40,000	0.119 (0.025–0.560)	0.007**	0.127 (0.017–0.944)	0.044**
	41,000–50,000	0.286 (0.053–1.539)	0.145	0.130 (0.013–1.327)	0.085
	>51,000	Ref		Ref	
History of conflict with partner	No	Ref		Ref	
	Yes	0.293 (0.173–0.498)	0.000**	0.216 (0.094–0.500)	0.000**
Gestational age	Postpartum			Ref	
	14–28 weeks	2.039 (0.984–4.227)	0.055	1.307 (0.486–3.511)	0.596
	29–40 weeks	0.105 (0.056–0.196)	0.000**	0.036 (0.013–0.098)	0.000**
	5–13 weeks	0.135 (0.071–0.257)	0.000**	0.097 (0.040–0.234)	0.000**
Problems in previous pregnancy	No	Ref		Ref	
	Yes	2.069 (1.352–3.165)	0.001**	2.851 (1.546–4.260)	0.001**
Smoking	No	Ref		Ref	
	Yes	2.360 (1.280–4.352)	0.006**	2.650 (1.038–6.771)	0.042**
Alcohol	No	Ref		Ref	
	Yes	0.137 (0.079–0.240)	0.000**	0.082 (0.035–0.193)	0.000**
Status disclosure	No	Ref		Ref	
	Yes	0.621 (0.405–0.953)	0.029**	0.523 (0.312–0.878)	0.014**

**Values significant at a *p* < 0.05.

Similarly, the factors associated with stress among WLHIV in Nigeria were assessed, and some of the statistically significant factors with stress among WLHIV in Nigeria at the crude OR remained significant after adjusting for potential confounders (income level, history of conflict with a partner, early and late gestational age, alcohol usage, and status disclosure). Women who have disclosed their status to their partners are about two times more likely to develop stress (OR = 2.094, CI = 1.191–3.682), while alcohol was observed to be a protective factor to the development of stress among WLHIV (OR = 0.357; CI = 0.170–0.752), early and late gestational age as well was observed to be a protective factor for the development of stress among WLHIV (OR = 0.158; CI = 0.070–0.357) and (OR = 0.049; CI = 0.019–0.126) respectively (see Table 6).

**Table 6:** Factors associated with stress among women living with HIV.

Variables	Category	Crude OR 95%CL (lower-upper)	*p*-value	Adjusted OR 95% CI (lower-upper)	*p* value
Age	19–24	2.923 (0.797–10.723)	0.106	2.927 (0.643–13.325)	0.165
	25–30	1.303 (0.678–2.502)	0.427	1.773 (0.746–4.215)	0.195
	31–36	1.487 (0.803–2.754)	0.207	1.448 (0.651–3.222)	0.364
	37–42	1.510 (0.819–2.786)	0.187	1.520 (0.689–3.353)	0.299
	43–49	Ref		Ref	
Income level	<20,000	0.158 (0.035–0.712)	0.016**	0.034 (0.006–0.199)	0.000**
	20,000–30,000	0.094 (0.019–0.459)	0.004**	0.028 (0.004–0.185)	0.000**
	31,000–40,000	0.069 (0.014–0.326)	0.001**	0.052 (0.009–0.302)	0.001**
	41,000–50,000	0.179 (0.034–0.944)	0.043**	0.098 (0.014–0.679)	0.019**
	>51,000	Ref		Ref	
History of conflict with partner	No	Ref			
	Yes	0.310 (0.176–0.546)	0.000**	0.423 (0.202–0.886)	0.023**
Gestational age	Postpartum	Ref		Ref	
	14–28 weeks	1.574 (0.915–2.709)	0.101	0.774 (0.377–1.589)	0.485
	29–40 weeks	0.128 (0.067–0.247)	0.000**	0.049 (0.019–0.126)	0.000**
	5–13 weeks	0.166 (0.085–0.324)	0.000**	0.158 (0.070–0.357)	0.000**
Problems in previous pregnancy	No	Ref		Ref	
	Yes	2.061 (1.381–3.077)	0.000**	1.722 (0.938–3.163)	0.080
Smoking	No	Ref		Ref	
	Yes	1.734 (1.026–2.930)	0.040**	1.750 (0.878–3.490)	0.112
Alcohol	No	Ref			
	Yes	0.290 (0.167–0.504)	0.000**	0.357 (0.170–0.752)	0.007**
Status disclosure	No	Ref			
	Yes	0.447 (0.297–0.674)	0.000**	2.094 (1.191–3.682)	0.010**

**Values significant at a *p* < 0.05.

Among the several factors identified for stress and depression among WLHIV, income level, history of conflict with a partner, early and late gestational age, alcohol usage, and status disclosure were significant factors associated with stress and depression. After adjusting for other factors, only three were reported to remain significant for comorbid depression and stress among WLHIV, including early and late gestational age, problems with previous pregnancy, and status disclosure (see Table 7).

**Table 7:** Factors associated with comorbid stress and depression among women living with HIV.

Variables	Category	Crude OR 95% CL (lower-upper)	*p*-value	Adjusted OR 95% CI (lower-upper)	*p* value
Problems in Previous Pregnancy	No	Ref		Ref	
	Yes	11.892 (5.475–25.831)	0.000**	7.034 (2.772–17.848)	0.000**
Alcohol	No	Ref	–	–	–
	Yes	0.183 (0.056–0.602)	0.005**	–	NS
Status disclosure	No	Ref		Ref	
	Yes	0.155 (0.083–0.290)	0.000**	0.269 (0.127–0.568)	0.010**
Gestational age	Postpartum	Ref		Ref	
	14–28 weeks	17.203 (7.367–40.170)	0.000**	7.034 (2.772–17.848)	0.000**
	29–40 weeks	0.224 (0.027–1.857)	0.166	0.161 (0.019–1.370)	0.095
	5–13 weeks	0.235 (0.277–0.033)	0.235	0.381 (0.044–3.294)	0.380

**Values significant at a *p* < 0.05; NS, not significant.

## Discussion

This study determined the prevalence and factors associated with depression and psychological stress among WLHIV during their perinatal period in Ibadan, Nigeria. The study results show a prevalence of 63.9 and 79.9% for depression and stress, respectively, among the respondents (20, 33). The prevalence of perinatal depression is higher than 38.4% in a similar study in Ethiopia (21). The prevalence of antenatal depression of 61.6% as measured by the EPDS with a cut-off ≥13 is slightly higher than the prevalence found in a previous study in Ekiti State, Nigeria (49.5%) (27), 47.6% in Addis Ababa, Ethiopia (21), and 52.5% in India among women on ART (29). The differences in prevalence might be due to differences in sociodemographic characteristics and tools used to assess depression. However, this finding buttresses the need to integrate mental health services into routine HIV care services, especially among women, to mitigate the adverse associated with maternal and child outcomes.

In the current study, the mean perceived stress was 12.27 among the pregnant and 14.41 among the non-pregnant respondents. This incident indicates moderate stress among WLHIV during the perinatal period. This finding is consistent with what was reported in another study in Nigeria, in which the mean perceived stress was moderate among the study population (31). The level of stress among the participants plausible predisposes to higher risks of mental disorders as it is in the general population and settings with social inequalities (32). Stress is a significant risk factor for depressive symptoms (34). Similar to other studies, it was found that women within the study sample who reported depressive symptoms (64.0%) reported significantly higher levels of perceived stress than women without depressive symptoms (28.0%).

This study used multivariate analysis to highlight factors associated with perinatal depression and perceived stress in a sample of WLHIV recruited from ART clinics. The study found that the status of the participants’ partners was significantly associated with depression and perceived stress. According to the study, participants who earned below 20,000 NGN were 5.6 times more likely to report symptoms of depression and perceived stress. The results are consistent with those reported in studies in Ethiopia and South Africa, which presented that low income and unemployment were related to depression among HIV-positive women (24, 35). The reason could be that in low-income countries, women are pressured to default academics for poverty-related factors, resulting in more prominent engagement in domestic work and the lack of access to health education and awareness. This factor is ascribed to the possible negative interaction between mental disorders (e.g., depression) and poverty, primarily because, in principle, people with depression commonly perform poorly in their daily tasks (36). In addition, pregnancy may decrease their employability and even their potential to work (21) because of the impoverished labor women may need to undertake (37).

This study also revealed that having a problem with previous pregnancy was a risk factor for depression among WLHIV. According to the study, pregnant women within their first (5–13 weeks) and third (29–40 weeks) trimesters have lower chances of developing depression. In contrast to other research that has found no link between gestational age and depression among women with HIV, our findings suggest that physiological changes during this stage may contribute to the onset of depression. Alternatively, the increased anxiety that women experience in the third trimester could also play a role (38, 39). This study indicates that problems in a previous pregnancy were significantly associated with the co-occurrence of depression and perceived stress. Our findings suggest that WLHIV who experienced complications in their previous pregnancy are twice as likely to report symptoms of depression and perceived stress compared to those who did not have such complications. This result could be because their complications were particularly severe and stressful for them. This result is consistent with previous studies highlighting a link between prior pregnancy complications and an increased risk of depression (40).

The prevalence of respondents with suicidal thoughts was examined, and the results indicate significant differences between those without depressive symptoms and those with depressive symptoms. Among respondents without depressive symptoms, a small proportion reported never having suicidal thoughts, while the majority experienced suicidal thoughts quite often. These findings are related to other studies conducted in Ethiopia (33), United States (41) and South Africa (42), which highlight the variability in the prevalence of suicidal thoughts among individuals with and without depressive symptoms. Most respondents with depressive symptoms did not report experiencing suicidal thoughts, which suggests that suicidal ideation may not be as prevalent in this population as anticipated (41, 42).

Further research is needed to understand the factors influencing the presence and severity of suicidal thoughts in individuals with depressive symptoms. Qualitative studies could provide valuable insights into the subjective experiences and contextual factors contributing to suicidal ideation. Such information would be crucial for developing targeted interventions and support systems to address the mental health needs of this population.

The study has several limitations that should be taken into consideration. Firstly, the sample used in the study was recruited from ART clinics, which may introduce sampling bias and limit the generalizability of the findings. The results may not represent all WLHIV in the general population who may not receive care at these clinics or be on ART. Secondly, the study design employed was cross-sectional, which hinders the establishment of causal relationships between the variables under investigation. A longitudinal study design would have been more suitable to determine the temporal relationship between perinatal depression, perceived stress, and associated factors. Thirdly, the study relied on self-report measures to assess depressive symptoms and perceived stress, which are subjective and prone to recall or social desirability bias.

Moreover, the study’s findings may have limited generalizability as it was conducted in a specific setting, namely Ibadan, Nigeria. Cultural, social, and economic factors unique to this context may have influenced the prevalence of depression and stress among WLHIV. Thus, caution should be exercised when applying these findings to other regions or countries. Lastly, the study solely relied on quantitative data, neglecting qualitative data that could have provided a deeper understanding of the experiences and perceptions of WLHIV regarding perinatal depression and stress. Incorporating qualitative data would have provided richer insights into the lived experiences of WLHIV and the contextual factors contributing to mental health outcomes.

Addressing these limitations in future research would enhance the findings’ validity, generalizability, and comprehensiveness, enabling a more nuanced understanding of perinatal depression and stress among WLHIV.

## Conclusion

This study revealed a high prevalence of depressive symptoms among WLHIV during their perinatal period. Low income, previous pregnancy complications, and gestational stage were associated with depression and perceived stress. The findings highlight the need for integrating mental health services into routine HIV care to address the adverse outcomes associated with maternal and child health. Additionally, while the prevalence of suicidal thoughts was relatively low among respondents with depressive symptoms, further research is warranted to understand the complex interplay between depression and suicidal ideation. Comprehensive assessments and targeted interventions are essential to address the mental health needs of women living with HIV during the perinatal period.

## Data availability statement

The original contributions presented in the study are included in the article/supplementary material, further inquiries can be directed to the corresponding author.

## Ethics statement

This study involving humans was conducted following the ethical principles of the Declaration of Helsinki. The study was approved by Ethics Committee of Lead City University, Ibadan (protocol code: LCU-REC/22/125) as well as from the Oyo State Ministry of Health Research Ethics Committee (protocol code: AD 13/479/44539). The study was conducted in accordance with the local legislation and institutional requirements. The participants provided their written informed consent to participate in this study. Written informed consent was obtained from the individuals for the publication of any potentially identifiable images or data included in this article.

## Author contributions

FA: Conceptualization, Data curation, Formal analysis, Funding acquisition, Investigation, Methodology, Project administration, Resources, Supervision, Visualization, Writing – original draft, Writing – review & editing. OA: Data curation, Formal analysis, Investigation, Methodology, Project administration, Writing – original draft, Writing – review & editing. AL: Data curation, Formal analysis, Investigation, Methodology, Writing – original draft. SB: Data curation, Formal analysis, Investigation, Methodology, Project administration, Writing – original draft. ZA: Data curation, Methodology, Writing – original draft. IA: Data curation, Methodology, Writing – original draft. MO: Data curation, Investigation, Methodology, Writing – original draft, Writing – review & editing. OO: Investigation, Methodology, Writing – original draft. AD: Data curation, Formal analysis, Investigation, Methodology, Writing – original draft. RS-A: Writing – original draft. AG: Data curation, Investigation, Methodology, Writing – original draft. DN: Data curation, Formal analysis, Methodology, Writing – original draft. AS: Writing – review & editing, Data curation, Investigation, Methodology, Writing – original draft. OE: Conceptualization, Data curation, Investigation, Validation, Visualization, Writing – review & editing.
